# Targeting EZH2 regulates tumor growth and apoptosis through modulating mitochondria dependent cell-death pathway in HNSCC

**DOI:** 10.18632/oncotarget.5606

**Published:** 2015-09-10

**Authors:** Xuan Zhou, Yu Ren, Lingping Kong, Guoshuai Cai, Shanshan Sun, Wangzhao Song, Yu Wang, Rui Jin, Lisha Qi, Mei Mei, Xudong Wang, Chunsheng Kang, Min Li, Lun Zhang

**Affiliations:** ^1^ Department of Maxillofacial and Otorhinolaryngology Oncology, Tianjin Medical University Cancer Institute and Hospital, Key Laboratory of Cancer Prevention and Therapy, Tianjin Cancer Institute, National Clinical Research Center of Cancer, Tianjin, China; ^2^ Tianjin Research Center of Basic Medical Science, Tianjin Medical University, Tianjin, China; ^3^ Department of Genetics, Geisel School of Medicine, Dartmouth College, Hanover, NH, USA; ^4^ Department of Pathology, Tianjin Medical University Cancer Institute and Hospital, Tianjin, China; ^5^ Department of Neurosurgery, Tianjin Medical University General Hospital, Laboratory of Neuro-Oncology, Tianjin Neurological Institute, Tianjin, China; ^6^ Department of Surgery, College of Medicine, The University of Oklahoma Health Sciences Center, Oklahoma City, OK, USA

**Keywords:** EZH2 (enhancer of zeste homolog 2), MICU-1 (mitochondrial calcium uptake 1), H3K27me3, apoptosis, head and neck squamous cell carcinoma (HNSCC)

## Abstract

EZH2 is a negative prognostic factor and is overexpressed or activated in most human cancers including head and neck squamous cell carcinoma (HNSCC). Analysis of The Cancer Genome Atlas (TCGA) HNSCC data indicated that EZH2 over-expression was associated with high tumor grade and conferred poor prognosis. EZH2 inhibition triggered cell apoptosis, cell cycle arrest and decreased cell growth *in vitro*. MICU1 (mitochondrial calcium uptake1) was shown to be down regulated when EZH2 expression was inhibited in HNSCC. When the EZH2 and MICU1 were inhibited, HNSCC cells became susceptible to cell cycle arrest and apoptosis. Mitochondrial membrane potential and cytosolic Ca^2+^ concentration analysis suggested that EZH2 and MICU1 were required to maintain mitochondrial membrane potential stability. A xenograft tumor model was used to confirm that EZH2 depletion inhibited HNSCC cell growth and induced tumor cell apoptosis. In summary, EZH2 is a potential anti-tumor target in HNSCC.

## INTRODUCTION

Head and neck squamous cell carcinoma (HNSCC) is the six common cancers of human beings [1]. Although a systemic treatment strategy is practiced in treating the patients with HNSCC, the overall survival remains low [2–4]. There is an urgent need to better understand the molecular mechanism and find a more affective molecular target with diagnosis and therapeutic potential in HNSCC.

Enhancer of zeste homolog 2 (EZH2), a catalytic component of Polycomb repressive complex 2 (PRC2), involved in the regulation of homeotic(Hox) gene expression and in the early steps of X-chromosome inactivation [5, 6]. Mounting evidence demonstrated that EZH2 function as an oncogene and a negative prognosis factor involving in carcinogenesis in many human cancer types [7–11]. EZH2 contains a SET domain with histone methyltransferases activity, which catalyses trimethylation of lysine 27 in histone 3 (H3K27me3) [12]. EZH2 and the other three core accessory proteins (EED, SUZ12, and RbAp46/48) induce chromatin compaction and consequently prevent transcription of target genes [12, 13].

In most cancer types, elevated EZH2 function has predominantly been associated with tumor cell proliferation, cell cycle and migration [14–17]. EZH2 mediated tumor suppressor genes’ silencing contributes to cancer progression. P21, p16 and NLK are validated to be direct targets of EZH2 [14, 18]. In human HNSCC cells, loss of EZH2 partially interfered with proliferation and invasion ability *in vitro* and *in vivo*. However, whether and how targets EZH2 inducing cell apoptosis is still missing.

Apoptosis is a cellular procedure that is regulated by different regulatory molecules and the dysfunction of apoptosis results in various pathological disorders in humans such as cancers [19]. Escape from apoptosis is a key attribute of tumor cells and facilitates proliferation. The mitochondrial dependent cell death pathway integrates stress and survival signaling to govern whether a cancer cell will live or die [20]. It is characterized by the loss of mitochondrial membrane potential(ΔΨm) [21]. Mitochondrial calcium uniporter (MCU), a notable mitochondrial Ca^2+^ uniporter complex, has been demonstrated to be regulated through its subunit mitochondrial calcium uptake 1(MICU1) in human models [22–24].

In the present work, we first analyzed EZH2 expression in a Chinese HNSCC cohort and HNSCC cohort form TCGA, and we further identify inhibition of EZH2 inducing HNSCC cell apoptosis in cell lines *in vitro* and in a Cal27 derived tumor model. We determined that mitochondrial dependent apoptosis in HNSCC cell was significantly induced when EZH2 was depleted and targeting EZH2 might represent a powerful strategy for the development of novel therapies interfering with distinct aspects of HNSCC.

## RESULTS

### EZH2 was highly expressed in HNSCC and conferred to poor patient survival

**Figure 1:**
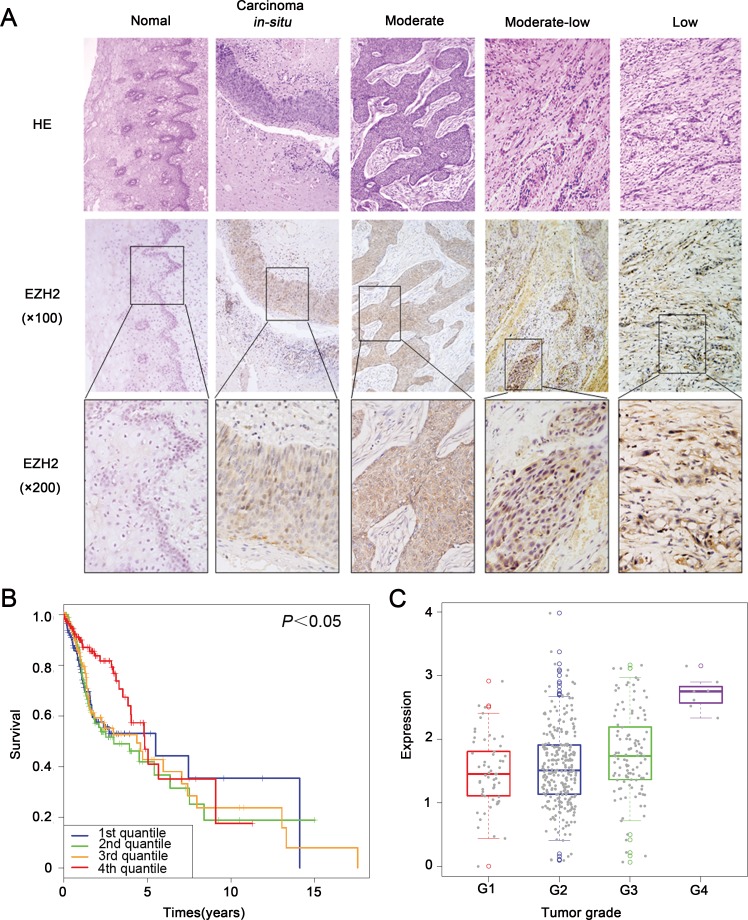
EZH2 was highly expressed in HNSCC and conferred to poor patient survival **A.** Representative images of immunohistochemical staining of EZH2 from human HNSCC. **B.** TCGA data analysis indicated that upper quantile EZH2 expression showed shorter survival comparing with the rest patients (*P* < 0.05). **C.** EZH2 expression of different tumor grades displayed obvious difference, y axis showed the log10 RPKM of EZH2 in TCGA datasets.

First, we analyzed EZH2 expression in a Chinese HNSCC cohort of 97 cases and normal oral cavity mucosa samples of 16 cases by using IHC assay. 12 of 16 normal oral cavity mucosa samples showed negative staining of EZH2. In agree with previous studies, human HNSCC displayed positive expression of EZH2 in tumor cell nuclei (Figure 1A). There were 49 HNSCC samples showed positive expression of EZH2 and 48 negative (50.51%). No statistical significance of EZH2 expression was determined between groups with different age at diagnosis and sex status (Table 1). HNSCC with larger tumor size ( > 2cm) showed a higher positive rate of EZH2 comparing with the smaller tumor size group (≤2cm, χ^2^ = 7.980, *P* = 0.006). Similarly, EZH2 expression of TNM stage IV HNSCC was higher than that of TNM stage I-III tumors (χ^2^ = 8.743, *P* = 0.037). Apart from tumor size and clinical stage, EZH2 was differently expressed among the HNSCC samples with different histological types. EZH2 expression was lower in well or moderate differentiated HNSCC than in poorly differentiated tumors (χ^2^ = 11.587, *P* = 0.003) (Table 1). These data implied EZH2 is a potential marker with diagnosis potential in HNSCC.

**Table 1:** Correlation between EZH2 and clinical-pathologic characteristics of patients with HNSCC

Variant	Total	EZH2 expression		χ^2^	*P* Value
		Negative (%)	Positive (%)		
Age					
<50	16	8(50.0)	8(50.0)	0.100	0.789
≥50	81	44(54.3)	37(45.7)		
Sex					
Male	78	39(50.0)	39(50.0)	2.090	0.201
Female	19	13(68.4)	6(31.6)		
Tumor size					
≤2cm	36	26(72.2)	10(27.8)	7.980	0.006*
>2cm	61	26(42.6)	35(57.4)		
Histological differentiation					
Well differentiated	18	16(88.9)	2(11.1)	11.587	0.003*
Moderately differentiated	63	30(47.6)	33(52.4)		
Poorly differentiated	16	6(37.5)	10(62.5)		
Clinical stage					
TNM I	36	25(69.4)	11(30.6)	8.473	0.037*
TNM II	20	10(50.0)	10(50.0)		
TNM III	16	9(56.2)	7(43.8)		
TNM IV	25	8 (32.0)	17(68.0)		

* Significantly different.

Second, to further study the signiﬁcance of high EZH2 expression for prognosis in HNSCC patients, we established four EZH2 status patient groups by using quantile based on RNAseq form The Cancer Genome Atlas (TCGA). Kaplan-Meier survival analysis showed that the patients with upper quantile EZH2 expression showed shorter survival comparing with the rest patients (*P* < 0.05; Figure 1B). EZH2 expression of different tumor grades displayed obvious difference (*P* < 0.05; Figure 1C).

### Targeting EZH2 suppressed its function in HNSCC cells

**Figure 2:**
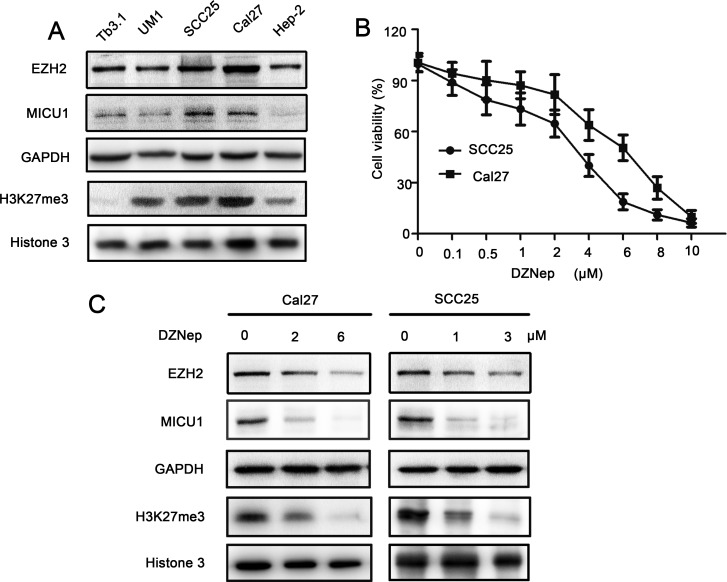
DZNep suppressed EZH2 function in HNSCC cell **A.** EZH2, MICU1 and H3K27me3 expression were determined by western blot analysis in five HNSCC cell lines. **B.** The cell viability curve of Cal27 and SCC25 cell lines. **C.** Western blot analysis of Cal27 and SCC25 cells shows the expression of EZH2, MICU1 and H3K27me3 after treatment with DZNep at 48 h, with GAPDH and Histone 3 serving as loading control.

Cal27 and SCC25 cells showed higher expression of EZH2, H3K27me3 and MICU1 comparing with Tb3.1, UM1 and Hep-2 cell lines (Figure 2A). To address EZH2's role in HNSCC, we blocked EZH2 activity in human HNSCC by chemical inhibition using DZNep. Cell viability curve indicated that, comparing with Cal27 cell (IC50 = 6μM), SCC25 (IC50 = 3μM) was more sensitive to DZNep (Figure 2B). DZNep treatment led to considerable reduction of EZH2, H3K27me3 and MICU1 expression in a dose-dependent manner (Figure 2C). Moreover, we employed EZH2 siRNA (si-EZH2) to block EZH2, the results showed that the expression of EZH2 and MICU1 were decreased (Figure S2A).

### EZH2 was required for growth of HNSCC *in vitro*

Since our previous report indicated that EZH2 inhibition suppressed glioma cell growth by regulating cell cycle progression, we hypothesized that there was a similar phenomenon in HNSCC. We employed several *in vitro* assays to demonstrate the requirement of EZH2 for HNSCC growth.

**Figure 3:**
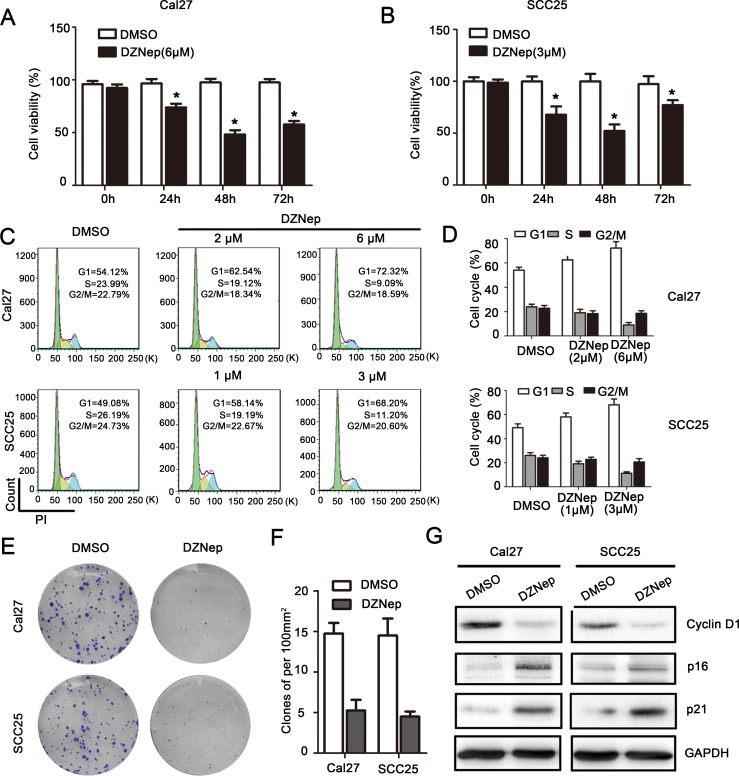
EZH2 was required for growth of HNSCC *in vitro* **A.**, **B.** Cal27 and SCC25 cells proliferation were significantly impaired after DZNep treatment for 48h as measured by MTT assay (* represent *P* < 0.05). **C.**, **D.** Flow-cytometry was performed to examine the G1/S arrest effect in Cal27 and SCC25 cells after treatment with DZNep at 48h (*P* < 0.05). **E.**, **F.** Reduction of clone formation ability in DZNep- treated Cal27 (6 μmol/L) and SCC25 (3μmol/L) cells (*P* < 0.05). **G.** Western blot analysis shows the expression of Cyclin D1, p16, p21 in both cells treated with DZNep (Cal27: 6μmol/L and SCC25: 3μmol/L) at 48h, with GAPDH as a loading control (*P* < 0.05).

MTT assay indicated that, DZNep treated Cal27 and SCC25 cell showed significantly reduction of cell viability comparing with DMSO treated cells at 24h, 48h and 72h (*P* < 0.05, Figure 3A, 3B), and the most significant reduction of cell viability is 48h after DZNep treatment. Flow-cytometry data revealed that significant G1 phase increase was observed in EZH2 treated Cal27 (1.16-1.34 folds) and SCC25 (1.18-1.39 folds) cells (*P* < 0.05, Figure 3C, 3D). Clone formation assay indicated that, 15 days after a single does-treatment of DZNep, the clones density of Cal27 reduced from (14.8 ± 2.6) to (5.3 ± 2.6) (per 100mm^2^) (*P* < 0.05), and the clones density of SCC25 reduced from (14.5 ± 4.2) to (4.5 ± 1.3) (per 100mm^2^) (*P* < 0.05, Figure 3E, 3F). The cell cycle dependent oncogene Cyclin D1 level was down-regulated while p16 and p21 expression were up-regulated by EZH2 blockage (Figure 3G).

### Targeting EZH2 induced apoptosis of HNSCC *in vitro*

**Figure 4:**
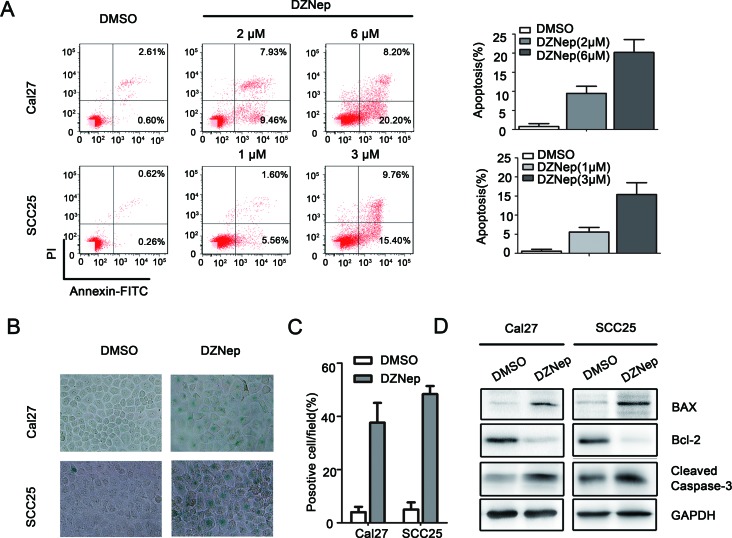
Targeting EZH2 induced apoptosis of HNSCC *in vitro* **A.** The percentages of apoptotic cells were significantly increased by DZNep treatment in a dose dependent (*P* < 0.05). **B.**, **C.** SA-β-gal staining positive cells were determined and compared after Cal27 and SCC25 cells were treated with DZNep (Cal27: 6μmol/L and SCC25: 3μmol/L) or DMSO for 48h (*P* < 0.05). **D.** Western blot analysis shows the expression of BAX, Bcl-2, Cleaved caspase-3 in both cells treated with DZNep (Cal27: 6μmol/L and SCC25: 3μmol/L) at 48h, with GAPDH as a loading control (*P* < 0.05).

Anti-apoptosis is the common feature of cancer progression. We employed Annexin V/PI assay to evaluate the anti-apoptosis role of DZNep or si-EZH2 in treating HNSCC. DZNEP induced early and latent phase of apoptosis in Cal27 (DMSO: 0.6%, DZNep (2μM: 9.5%, 6μM: 20.2%), *P* < 0.05) and SCC25 (DMSO: 0.3%, DZNep (1μM: 5.6%, 3μM: 15.4%), *P* < 0.05, Figure 4A) cell line, and si-EZH2 also induced early and latent phase of apoptosis in two cell lines. To assess the effect of EZH2 inhibition in inducing cell senescence, senescence-related β-galactosidase staining is employed. In contrast to DMSO treated cells, DZNep treated Cal27 and SCC25 cells displayed a 9- to 10-fold (*P <* 0.05) higher SA-β-Gal activity in both cell cultures (Figure 4B, 4C). Similarly, si-EZH2 increased SA-β-Gal activity in two cell lines (Figure S2D, E). We then analyzed the changes in the levels of pro-apoptotic proteins BAX, and Cleaved caspase-3, and anti-apoptotic protein Bcl-2. The results indicated that Bcl-2 was decreased and BAX or Cleaved caspase-3 was increased by DZNep or si-EZH2 treatment in both cell lines (Figure 4D, S2F).

**Figure 5:**
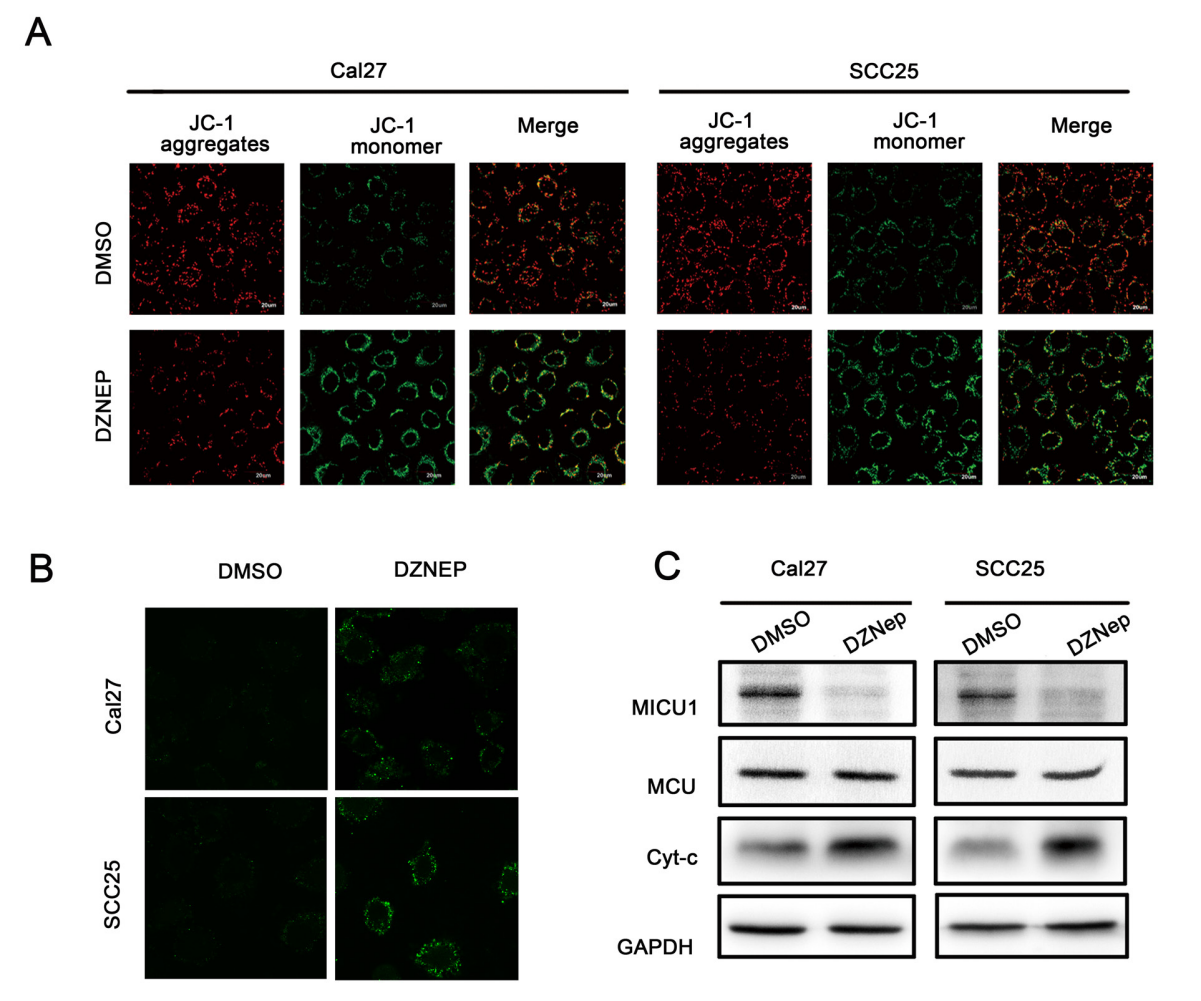
Targeting EZH2 affected mitochondrial membrane potential balance **A.** Mitochondrial membrane potentials were reduced by DZNep (Cal27: 6μmol/L and SCC25: 3μmol/L), the evident in confocal image soft the fluorescent dye JC-1 in Cal27 and SCC25 cell lines. **B.** Ca^2+^ indicator to determine the free Ca^2+^ level induced by DZNep (Cal27: 6μmol/L and SCC25: 3μmol/L). **C.** Western blot analysis shows the expression of Cytochrome c, MICU1, MCU in both cells treated with DZNep (Cal27: 6μmol/L and SCC25: 3μmol/L) at 48h, with GAPDH as a loading control (*P* < 0.05).

Moreover, the loss of ΔΨm is an early event in apoptosis. ΔΨm was detected by JC-1 fluorescent probe. As shown in Figure 5A, when Cal27 and SCC25 cell were treated with DZNep for 24h, ΔΨm was drastically reduced respectively in both cell lines. We measured ΔΨm after Cal27 and SCC25 cell were transfected with si-EZH2, the result showed that si-EZH2 reduced ΔΨm in both cell lines (Figure S2C). Next we used Ca^2+^ indicator, Fluo 3-AM, to determine the free Ca^2+^ level induced by EZH2 inhibition. The results showed that DZNep treatment of Cal27 and SCC25 cell increased Ca^2+^ accumulation in cytoplasm (Figure 5B). More importantly, DZNep treatment inhibited the cyto-Ca^2+^regulator MICU1 expression in both cell lines. MCU expression showed no significant changes by DZNep treatment. By contrast, Cytochrome c expression was increased dramatically (Figure 5C).

Thirdly, we investigated the correlations between EZH2 and other apoptosis related genes by using linear regression association test in HNSCC dataset published by TCGA. A gene with p-value less than 0.05 was considered to be significantly EZH2-associated. We observed that EZH2 was positively associated with CBARA1 (MICU1), BCL2 and MKI67, and negatively associated with CASP4, CCND1 and CDKN1A in the term of expression (P < 0.05, see Figure S1A–F).

### MICU1 regulated HNSCC cell apoptosis *in vitro*

**Figure 6:**
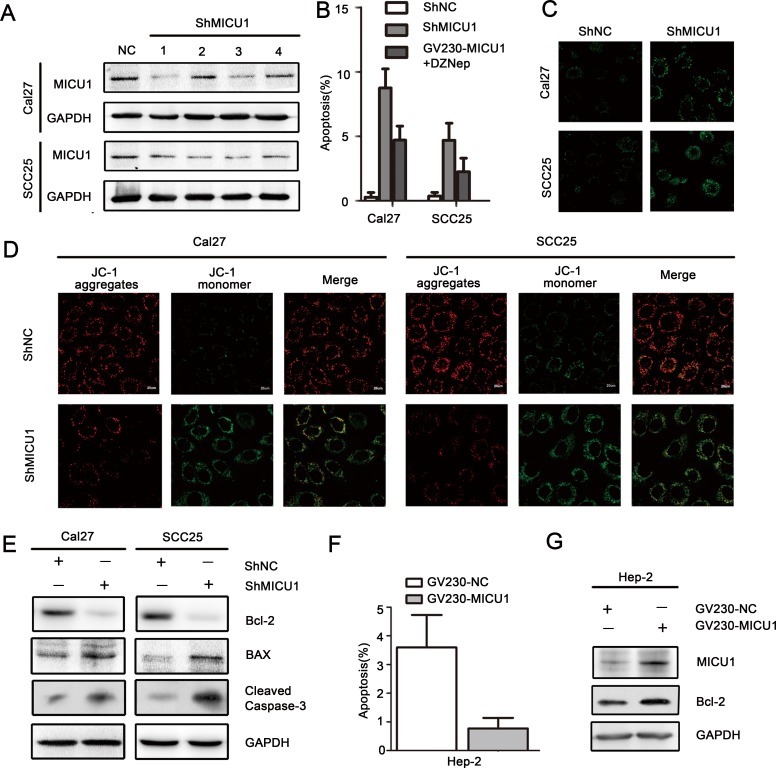
MICU1 regulated HNSCC cell apoptosis *in vitro* **A.** MICU1 expression changes induced by shMICU1 in both cell lines. **B.**: Flow cytometry was performed to examine the apoptosis of Cal27 and SCC25 cells after transfection with ShMICU1 or GV230-MICU1 combining with DZNep (Cal27: 6μmol/L and SCC25: 3μmol/L). **C.** Ca^2+^ indicator to determine the free Ca^2+^ level induced by MICU1 inhibition. **D.** Mitochondrial membrane potentials were reduced by MICU1 inhibition, the evident in confocal image soft the fluorescent dye JC-1 in Cal27 and SCC25 cell lines. **E.** Western blot analysis shows the expression of BAX, Bcl-2, Cleaved caspase-3 in both cells treated with ShMICU1 at 48h, with GAPDH as a loading control (*P* < 0.05). **F.** Flow-cytometry was performed to examine the apoptosis of Hep-2 after transfection with GV230-MICU1. **G.** Western blot analysis shows the expression of MICU1, Bcl-2 in Hep-2 cell treated with GV230-MICU1 at 48h, with GAPDH as a loading control (*P* < 0.05).

Next, we employed a MICU1 shRNA (shMICU1) vector to further validate the role of targeting EZH2/MICU1 in regulating cell apoptosis in HNSCC cells. We first analyzed the MICU1 expression changes induced by shMICU1 in both cell lines, and shMICU1-3 treatment showed the robust effect in inhibiting MICU1 expression (Figure 6A). Thus, we selected shMICU1-3 for further research usage. The Annexin V/PI assay results indicated a significant increase in cell apoptosis with the depletion of MICU1, the ratio of early apoptotic cell increased from 0.28% to 8.75% in Cal27 and 0.18% to 4.66% in SCC25 (*P* < 0.05, Figure 6B). Moreover, we combined MICU1 expression vector (GV230-MICU1) with DZNEP treated two cell lines, the ratio of early apoptotic cell is 4.7% in Cal27 and 2.26% in SCC25 (*P* < 0.05, Figure 6B). As shown in Figure 6C, when Cal27 and SCC25 cell were treated with shMICU1 for 48h, ΔΨm was drastically reduced respectively in both cell lines and Ca^2+^ indicator suggested free Ca^2+^ level induced by MICU1 targeting (Figure 6C, 6D). Western blot analysis showed that Bcl-2 was decreased and BAX or Cleaved caspase-3 was increased by shMICU1 treatment in both cell lines (Figure 6E). Moreover, we introduced GV230-MICU1 into Hep-2 cell that had a low endogenous MICU1 expression level as shown in Figure 2A. Early apoptosis rate decreased from 3.6% to 0.8% in Hep-2 (Figure 6F). Western blot analysis showed that anti-apoptotic protein MICU1 and Bcl-2 was increased (Figure 6G). These data provide important evidence that MICU1 play an important role in EZH2- regulated cell death pathway.

### Targeting EZH2 inhibited HNSCC tumor growth and induced apoptosis *in vivo*

**Figure 7:**
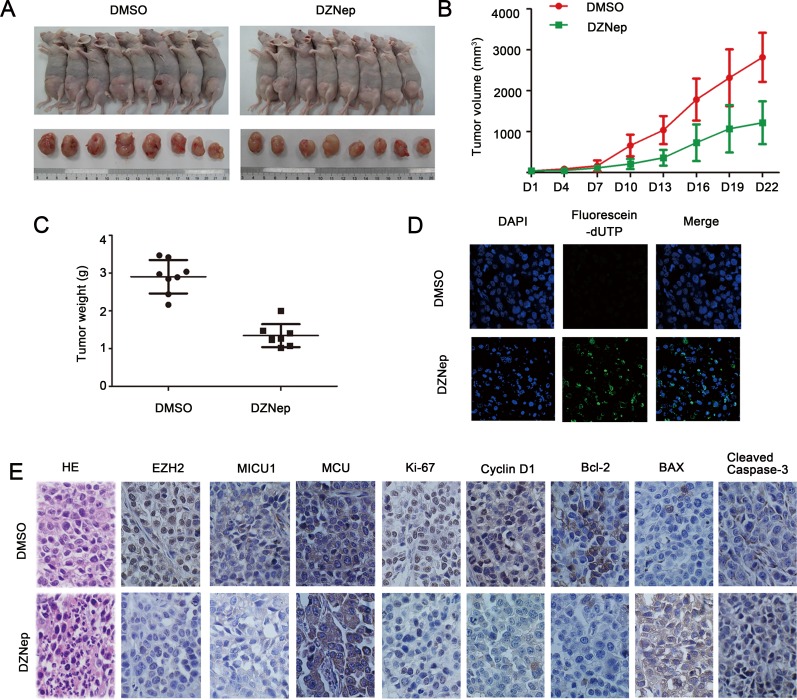
Targeting EZH2 inhibited HNSCC tumor growth and induced apoptosis *in vivo* **A.–C.** Tumor volume and weight of DMSO or DZNep-treated animals was measured. **D.** TUNEL assay showed an induced apoptotic nucleus (green) in DZNep-treated Cal27 tumors (magnification: x1000). **E.**: Representative images of immunohistochemical staining of EZH2, MICU1, MCU, Ki67, CyclinD1, Bcl-2, BAX and Cleaved caspase-3 in tissue from mice with tumors derived from Cal27 cells treated with DMSO or DZNep, and HE staining was used to examine morphological changes (magnification: 200x).

To better illustrate the role of EZH2 in HNSCC tumor growth, we established HNSCC xenograft tumor model using Cal27 cell lines. Comparing with DMSO treated tumors, DZNep treated tumors showed significant reduction of tumor volume and weight (*P* < 0.05, Figure 7A–7C). TUNEL assay was used to determine the DNA fragmentation, which was the hallmark of apoptotic cells. The results indicated that DZNep treatment induced more apoptotic nuclei than in DMSO treated Cal27 xenograft tumors (Figure 7D). Strikingly, HE staining showed morphological changes in Cal27 tumors by DZNep treatment. For example, in DZNep treated group, Cal27 tumors displayed less mitotic figures, chromosome staining and cell density (Figure 7E). Most importantly, comparable to controlled tumors, DZNep treatment was sufficient to attenuate cell proliferation protein Ki-67, cyto-Ca^2+^ regulator MICU1, anti-apoptotic protein Bcl-2 as well as EZH2, and to increase Cleaved caspase-3 expression *in vivo* (Figure 7E).

## DISCUSSION

In this study, we demonstrate over-expressed EZH2 to be a key target with diagnosis and therapeutic potential in HNSCC. Analysis of HNSCC TCGA dataset suggested EZH2 activity might influence the downstream apoptosis related genes. Importantly, inhibition of EZH2 with chemical synthesized compound DZNep, induced loss of ΔΨm and cell apoptosis in HNSCC cell *in vitro* and *in vivo*. We also reported that EZH2 inhibition induced MICU1 inactivation and cyto-Ca^2+^ accumulation which might be the underling mechanism.

Elevated EZH2 levels often are directly correlated with advanced metastatic stages of cancer progression and poor prognosis. In glioma and renal cell carcinomas, increased EZH2 expression was associated with tumor grade and high expression of EZH2 was determined to be a strong and independent predictor of short overall survival [16, 25]. Comparing with normal mucosa or adjacent normal tissue, EZH2 was overexpressed in 50-60% of HNSCC tissues and contributed to tumor differentiation status [26, 27]. Additionally, EZH2 has been found to initiate oral leukoplakia malignant transformation and epithelial-mesenchymal transition in HNSCC [28]. In the present work, we analyzed the EZH2 expression in TCGA dataset, and we found the higher expression of EZH2 might predict shorter overall survival of HNSCC patients. And examination of HNSCC specimens showed EZH2 expression was associated with tumor size, histological differentiation and clinical stage. Our data further confirmed EZH2 was a robust prognostic factor for human HNSCC and a biomarker for identifying HNSCC molecular subtypes.

EZH2 is required for tumor progression in H3K27me3 dependent manner, and this can be blocked by EZH2 inhibitors, like GSK126 or EPZ-6438 [29]. In tongue cancer and glioblastoma, EZH2 depletion suppressed tumor growth by arresting cell cycle progression [17, 30]. Our data suggested that, we used pharmacological EZH2 inhibition via DZNep, which causes proteosomal degradation of EZH2 [31, 32]. DZNep treatment effectively reduced EZH2 and H3K27me3 expression in both cell lines. Of note, EZH2 inhibition impairs tumor cell proliferation ability, cell cycle progression and anti-apoptisis potential in both cell lines and this suppressed Cal27 derived tumors’ growth in a xenograft model. These data fully illustrated that EZH2 serves as a target with therapeutic potential in HNSCC.

To address the mechanism through which EZH2 inhibition effecting tumor cell apoptosis, we found that HNSCC cell showed spatially increase of cyto-Ca^2+^ and loss of ΔΨm was induced by DZNep treatment. A well-established notion that MCU is a highly selective channel with Ca^2+^ transmission capability and MICU1 has been shown to regulate the mitochondrial Ca^2+^uniporter, which pumps cytosolic Ca^2+^ into the mitochondria [33]. In the absence of MICU1, mitochondria become constitutively loaded with Ca^2+^, triggering excessive reactive oxygen species generation and sensitivity to apoptotic stress [23, 34]. Although the underlying mechanisms by which EZH2 regulates MICU1 to prevent HNSCC cell apoptosis is not entirely clear, our data showed inhibiting EZH2 triggers cyto-Ca^2+^ accumulation, loss of ΔΨm, G1 phase cell cycle arrest and changes on proteins of mitochondrial related cell death pathway. More importantly, gain or loss function assays suggested that targeting MICU1 could compensate EZH2's effect on regulating tumor cell apoptosis.

On the basis of increasing integrated research and experimental evidence, it is now fully elucidated that EZH2 plays a central role in cancer transformation, and its overexpression is associated with tumor cell anti-apoptosis, invasiveness, chemo-resistance in human cancers including HNSCC. Further, pharmacological inhibition of EZH2 by using DZNep, GSK503 [35], GSK126 [36] and etc provided a novel approach in treating human epithelium cancers. Accordingly, our study reveals that targeting EZH2 might be a promising strategy for future therapies of human HNSCC patients.

## MATERIALS AND METHODS

### HNSCC samples and Immunohistochemistry for EZH2

A total of 97 HNSCC tumor samples from radical resection were randomly collected at Tianjin Medical University Cancer Institute & Hospital during January 2009 to December 2012. The demographic and clinical data was collected from electric records. All the tissue samples were used for EZH2 expression examination. All samples were collected with informed consent according to the Human Tissue Sample Usage Guidelines of the Tianjin Medical University Medical Ethnic Committee.

Immunohistochemistry staining for EZH2 (Cell Signaling Technology) of 97 HNSCC samples from Tianjin Medical University Cancer Institute & Hospital was performed on formalin-fixed, paraffin-embedded tissue sections, according to the manufacturer's instructions. Each stained slide was jointly scored by 2 pathologists blinded to the clinical information. For statistical analysis, positive expression of EZH2 was defined as > 10% of positive-stained cells in the tumor. Two blinded pathologists independently evaluated the slides. In case of a discrepancy, the 2 observers simultaneously reviewed the slides to achieve a consensus.

### HNSCC mRNA datasets and analysis

Transcriptom expression datasets and the corresponding clinical information were downloaded from websites of The Cancer Genome Atlas (http://cancergenome.nih.gov). Total 508samples were available for this analysis. RPKM were calculated from RNA-seq read counts of each mRNA and log transformations were performed to increase the normality. We applied linear regression study the gene co-expression and ordinal data analysis to study the association between EZH2 expression and tumor stage information. With survival duration data from TCGA, we estimated Kaplan-Meier survival curves of low-risk and high risk groups separated by the mean of EZH2 expression. Also the Log-rank test were performed to test the significance of difference of survival curves between these two groups. Data manipulation, statistical analysis and visualization were accomplished using R 3.0.2.

### Cell lines and culture conditions

Human HNSCC cell line Hep-2 was purchased from the Institute of Basic Medical Sciences, Chinese Academy of Medical Sciences. Tb3.1 cell line was a gift from the Ninth People's Hospital Shanghai Jiao Tong University. Cal27, SCC25, UM1 cell lines were was kindly provided by Prof. JinsongHou of Guanghua School of Stomatology, Hospital of Stomatology, SunYat-sen University. The cell lines were maintained in MEM, DMEM or RPMI-1640 (Thermo Scientiﬁc) supplemented with 10% fetal bovine serum (FBS, Thermo Scientiﬁc) with 5% CO_2_ at 37°C.

### Drug treatments

For EZH2 inhibition, Cal27 and SCC25 cells were treated with 3-Deazaneplanocin A (DZNep) (2 or 6μmol/L for Cal27 cell, 1or 3μmol/L for SCC25 cell) (Merck Millipore) dissolved in dimethyl sulfoxide (DMSO, Sigma-Aldrich) for 48 hours.

### SiRNA, ShRNA and vector transfection

SiRNA (GeneChem) was used to knockdown EZH2 expression in Cal27 and SCC25 cell lines, using Lipofectamine 3000 reagent as the manufacturer's instructions (Life Technology). The final concentration of EZH2 siRNA is 75nM. 48h after transfection the Cal27 and SCC25 cell lines were harvest for research usage.

ShRNA (GeneChem) were used to knockdown MICU1 expression in Cal27 and SCC25 cell lines. 5μg shRNA was transfected to cells, using Lipofectamine 3000 reagent as the manufacturer's instructions (Life Technology).

5μg MICU1 full-length expression vector (GV230-MICU1, GeneChem) was transfected to 2×10^6^ HNSCC cells by using Lipofectamine3000 reagent as the manufacturer's instructions (Life Technology). The GV230 empty vector (GV230-NC, GeneChem) was used as the control plasmid. 48h after transfection, the cells were collected for apoptosis examination and whole cell lysate were collected for Western blot analysis.

### Cell experiments *in vitro*

For apoptosis analysis by flow cytometry, DMSO or DZNep treated Cal27 and SCC25 cells were digested with trypsin (Gibico) and resuspended as single-cell suspension. Double staining with fluorescein isothiocyanate (FITC)-Annexin V and propidium iodide (PI) was completed using a FITC Annexin V Apoptosis Detection Kit (Keygen) according to the instructions provided by the manufacturer. Apoptosis was measured using flow cytometry.

For cell cycle distribution analysis by flow cytometry, after DMSO or DZNep treated Cal27 and SCC25 cells, the cells were trypsinized, fixed in 70% ethanol, washed once with PBS, and then labeled with propidium iodide (Keygen) in the presence of RNase A (Sigma-Aldrich) for 30 min in the dark. Cell cycle distribution was measured using flow cytometry.

For colony forming assay, 1,000 cells treated with DZNep or DMSO were seeded into 6-well plates and allowed to grow for 14 days. The cells were then fixed and stained with crystal violet. The colonies were further visualized under an invert microscope and photographed. Colonies numbers were counted from five different fields per 100 mm^2^.

SA-β-gal staining (Beyotime Biotechnology) was performed following the supplier's instructions. SA-β-gal positive cells were stained in blue. Total 500 cells from five different vision fields per section were counted under a pathology light microscope (Olympus) at 200×. All of the stained sections were examined by two independent observers.

JC-1 probe (Beyotime Biotechnology) was employed to measure mitochondrial depolarization. Briefly, Cells cultured in confocal capsule after indicated treatments were incubated with JC-1 staining solution at 37°C for 20 min and rinsed twice with PBS. MMP were monitored by determining the relative amounts of dual emissions from mitochondrial JC-1 monomers or aggregates using FV-1000 laser scanning confocal biological microscopes (Olympus) under Argon-ion 488 nm and 546 nm laser excitation. Mitochondrial depolarization is indicated by an increase in the green/red fluorescence intensity ratio.

Ca^2+^ indicator Fluo-3/AM was used to detect the intracellular Ca^2+^. After 24 h, the treated cells and the control cells, rinsed twice with PBS, were loaded with theFluo-3/AM (5μM) and continuously cultured at 37°C for 60 min, at 5% CO_2_ incubator. The labeled cells were washed with PBS to remove the unloaded Fluo-3/AM. After cells were loaded with the fluorescence probe, changes in intracellular Ca^2+^of individual cells were measured using a digital imaging system equipped with a laser confocal scanning microscope (Olympus) with an excitation wavelength of 488 nm.

Western blot analysis was employed to assay protein expression. Total protein is extracted from DZNEP/shMICU1 treated HNSCC cells. The protein samples were resolved by SDS-PAGE and transferred onto PVDF membranes (Roche). The membranes were then incubated with the following antibodies: EZH2, MICU1, p16, p21, CyclinD1 and Cleaved caspase-3 (Cell Signaling Technology), MCU and Cytochrome c (Abcam), Bcl-2 and BAX (Zhongshan Biotechnology), H3K27me3 (Signalway Antibody). GAPDH (Zhongshan Biotechnology) or Histone 3 (Cell Signaling Technology) was used as an internal control.

### Cal27 tumor model study

Animal experiments were carried out using BALB/c-A nude mice at 4 weeks of age, which were purchased from Vital River Laboratories China. All experimental animals were injected subcutaneously on the right groin region with 2×10^6^ Cal27 cells. Twenty days later, tumors were formed, these mice were separated into two groups (8 mice per group), randomly. The DMSO group, the DZNep group (0.07 mg/kg dissolved in DMSO) [10]. The mice received an injection into the tumor every three days, and the size of tumor and mice weights were measured by calipers and electronic weighing meter. Tumor volume was calculated as follows: volume = a*b2/2. Tumor weights were also measured on tumor samples harvested.

### TUNEL assay

The cell apoptosis of the tumor samples was measured by TUNEL assay using an in situ cell death kit (Roche) by following the manufacture's protocol. The cell nucleuses were counterstained by DAPI (Sigma) reagent. Positive cells were visualized by FV-1000 laser scanning confocal biological microscopes (Olympus).

### Immunohistochemistry (IHC)

The paraffin-embedded tissue sections were used for examination of EZH2, MICU1, CyclinD1and Cleaved caspase-3 (Cell Signaling Technology), MCU (Abcam), Bcl-2, BAX, Ki-67 (Zhongshan Biotechnology) expressions. For IHC assay, sections were incubated with primary antibodies (1:100 dilutions) overnight at 4°C, followed by a biotin-labeled secondary antibody (1:100 dilutions) (Zhongshan Biotechnology) for 40 min at 37°C. Sections were incubated with ABC-peroxidase anddiaminobenzidine (DAB) (Zhongshan Biotechnology), counterstained with hematoxylin and visualized using light microscopy.

## SUPPLEMENTARY MATERIAL FIGURES


