# Supplementary material for: Targeting EZH2 regulates tumor growth and apoptosis through modulating mitochondria dependent cell-death pathway in HNSCC
Source: Oncotarget. 2015 Sep 10;6(32):33720–32. doi: 10.18632/oncotarget.5606 (PMC4741797; doi:10.18632/oncotarget.5606)
Supplement: Supplementary file 1 [file oncotarget-06-33720-s001.pdf]

Targeting EZH2 regulates tumor growth and apoptosis through modulating mitochondria dependent cell-death pathway in HNSCC

Supplementary Material

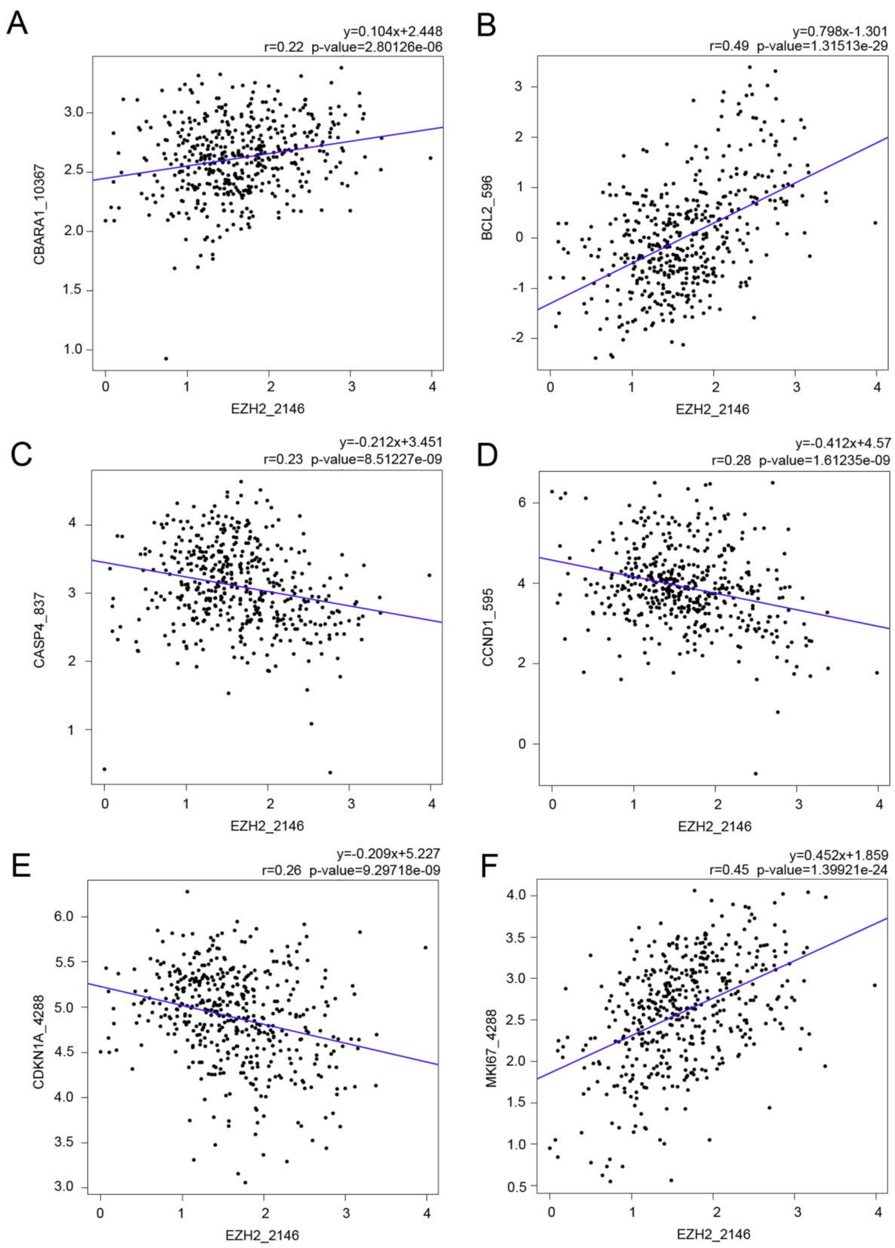

Supplementary Figure S1

EZH2 expression was significantly associated with apoptosis related genes(A, CBARA1; B, BCL2; C, CASP4; D, CCND1; E,CCKN1A; F,MKI67) in TCGA dataset(*P*<0.05).

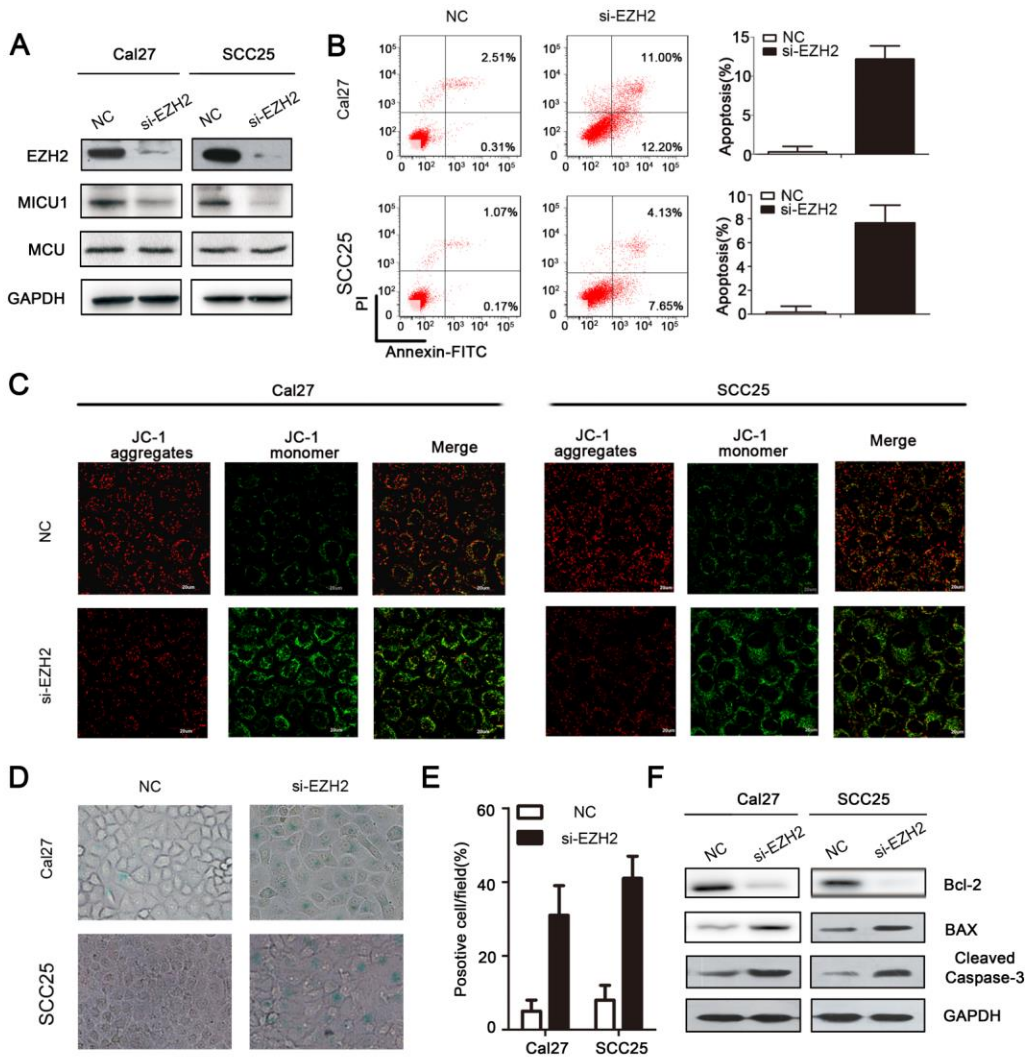

Supplementary Figure S2

Si-EZH2 induced cell apoptosis and inhibited tumor cell growth in HNSCC. (A) Western blot analysis of Cal27 and SCC25 cells shows the expression of EZH2, MICU1, MCU after treatment with si-EZH2 at 48 h, with GAPDH serving as loading control. (B) The percentages of apoptotic cells were significantly increased by si-EZH2 treatment (P<0.05). (C) Mitochondria membrane potentials were reduced by si-EZH2, the evident in confocal image soft the fluorescent dye JC-1 in Cal27 and SCC25 cell lines. (D, E) SA-β-gal staining positive cells were determined and compared after Cal27 and SCC25 cells were treated with si-EZH2 for 48h (P<0.05). (F) Western blot analysis shows the expression of BAX, Bcl-2, Cleaved caspase-3 in both cells treated with si-EZH2 at 48h, with GAPDH as a loading control (P<0.05).
